# Genotoxicity of Occupational Pesticide Exposures among Agricultural Workers in Arab Countries: A Systematic Review and Meta-Analysis

**DOI:** 10.3390/toxics11080663

**Published:** 2023-08-01

**Authors:** Moustafa Sherif, Khadija Ramadhan Makame, Linda Östlundh, Marilia Silva Paulo, Abderrahim Nemmar, Bassam R. Ali, Rami H. Al-Rifai, Károly Nagy, Balázs Ádám

**Affiliations:** 1 Institute of Public Health, College of Medicine and Health Sciences, United Arab Emirates University, Al Ain P.O. Box 15551, United Arab Emirates; 202190149@uaeu.ac.ae (M.S.); rrifai@uaeu.ac.ae (R.H.A.-R.); 2 Department of Public Health and Epidemiology, Faculty of Medicine, University of Debrecen, 4032 Debrecen, Hungary; khadija.makame@med.unideb.hu; 3 University Library, Örebro University, SE-702 81 Örebro, Sweden; linda.ostlundh@oru.se; 4 IPH, CHRC, NOVA Medical School, Faculdade de Ciências Médicas, NMS, FCM, Universidade Nova de Lisboa, 1169-056 Lisbon, Portugal; marilia.paulo@nms.unl.pt; 5 Department of Physiology, College of Medicine and Health Sciences, United Arab Emirates University, Al Ain P.O. Box 15551, United Arab Emirates; anemmar@uaeu.ac.ae; 6 Department of Genetics and Genomics, College of Medicine and Health Sciences, United Arab Emirates University, Al Ain P.O. Box 15551, United Arab Emirates; bassam.ali@uaeu.ac.ae

**Keywords:** pesticide, occupational exposure, agriculture, Arab countries, genotoxicity, DNA damage

## Abstract

Exposure to pesticides in Arab countries is a significant public health concern due to extensive agricultural activity and pesticide use. This systematic review aimed to evaluate the genotoxic effects of agricultural pesticide exposure in the region, identify research gaps, and assess methodological limitations. Following the PRISMA guidelines, a comprehensive search yielded five relevant studies conducted in Egypt, Syria, and Jordan. Various genotoxicity assays were employed, revealing a higher level of DNA damage in exposed compared to non-exposed individuals. Farmers exposed to pesticides exhibited a significantly higher occurrence of chromosomal translocation (t(14;18)), micronuclei, and chromosomal aberrations. However, only two studies assessed cytotoxicity indirectly. The studies predominantly focused on male participants, with variations in sample size and pesticide types. The lack of detailed exposure data necessitates cautious interpretation. This review underscores the need for further research on the genotoxicity of occupational pesticide exposure in the Middle East. Future studies should adopt robust study designs, collect biological and environmental samples, conduct repeated sampling, analyze seasonal variations, and encompass diverse study sites associated with specific crop groups.

## 1. Introduction

Pesticides are a class of agrochemicals that are widely employed worldwide to manage “pests” like bacteria, fungi, weeds, snails, insects, rodents, and worms. Pesticides are categorized based on their intended target species, such as insecticides, fungicides, herbicides, nematicides, rodenticides, acaricides, molluscicides, repellents, and growth regulators [1]. Pesticides encompass a high number of chemical substances that are marketed in various formulations. For instance, under the California Department of Pesticide Regulation, 1060 active ingredients are currently registered, which are marketed under an extensive array of 13,129 different product names [2]. Moreover, these pesticides are formulated in various forms, including liquids, concentrates, granules, powder, resin strips, impregnated pellet-tablets, and encapsulated particles [3]. In order to enhance the efficacy of pesticides and prevent the development of pesticide resistance, novel formulations are being constantly developed, which often contain various additional ingredients, such as surfactants and solvents. It is important to note that these added ingredients may also contribute to the toxicity of the pesticide product [4,5].

There are several ways in which humans may be exposed to pesticides, including inhalation of aerosols, dermal absorption during the mixing, loading, spraying, and harvesting of crops and livestock management, as well as by consumption of contaminated food or water [6]. Agricultural workers who handle these chemicals in agricultural settings are particularly at risk for acute and chronic toxicity [7].

The toxicity of pesticides can extend beyond their intended target species and affect non-target organisms due to the similarities in their basic biological processes [8]. This highlights the importance of considering the potential human health risks associated with pesticide exposure, particularly among those who work with these chemicals in agricultural settings. Exposure to pesticides may increase the risk of developing various diseases depending on the chemical properties of the pesticide, and the level and duration of exposure. Previous research has identified respiratory diseases, cancers, diabetes, immune toxicity, and neurodegenerative and neurodevelopmental disorders, among others, as potential health consequences associated with pesticide exposure [9]. 

Experimental studies have demonstrated that certain agrochemical substances, such as dichlorodiphenyltrichloroethane (DDT), are able to induce genotoxicity and mutagenicity, the precursors of carcinogenesis [10]. As a result, the International Agency for Research on Cancer (IARC) has identified certain pesticides, including diazinon, glyphosate, and malathion, as possible human carcinogens (group 2A) based on human epidemiological, animal, and in vitro studies [11,12]. In parallel, various pesticides have been banned or strictly restricted by regulatory bodies and legal instruments, such as the US EPA and the European Union’s Directive 91/414/EEC. In Europe, regulations pertaining to pesticides are primarily based on assessing the adverse effects of the active ingredients along with some representative formulations. However, these regulations may not always accurately assess the potential long-term effects of pesticide exposure, including genotoxicity and carcinogenicity, or consider the interactions between the active and other ingredients in pesticide formulations [13].

Biomonitoring assessment tools have been developed to regulate and protect populations exposed to such risks more effectively. In addition to assessing the internal dose of xenobiotics, some of these assays also serve as early indicators of altered structure and function (biological effect monitoring) and can be used to investigate populations exposed to pesticides. Genotoxicological tests aim to measure various genetic endpoints, including gene mutations, chromosomal aberrations, and direct DNA damage, which has been reviewed previously in the literature [9,14,15].

To our knowledge, no systematic review and synthesis of biological effect monitoring data on occupational pesticide-exposure-induced DNA damage among agricultural workers has yet been conducted in Arab countries. Agricultural workers in Arab countries often work and live in substandard conditions where protective measures, including personal protective equipment (PPE), are rarely used during pesticide application. The need to synthesize existing information is substantially justified if we also take into account the possibility of lax attitude in Arab nations toward the registration of pesticides [16], in addition to the poor enforcement of legislation that is meant to protect workers as well as the general population.

The aim of this study is to conduct a rigorous and thorough systematic review of peer-reviewed literature on biomonitoring studies from Arab countries that investigate the genotoxic effects of occupational pesticide exposure in agricultural workers, a population at high risk. The objective is to collect information on pesticide-induced DNA damage and identify knowledge gaps that may assist in the development of effective preventive measures.

## 2. Materials and Methods

### 2.1. Review Objectives

Our primary objective was to assess the magnitude of genotoxicity and association between pesticide exposure and DNA damage among agricultural workers in Arab countries who are occupationally exposed to pesticides. Additionally, this research aims to identify potential risk factors associated with genotoxicity in this population. To achieve these objectives, a Population, Exposure, Comparator, Outcome (PECO) statement has been formulated as a framework for the study (Table 1) [17].

### 2.2. Identification and Management of Studies

The study protocol was made available as a preprint on the medRxiv platform [18], and registered in the International Prospective Register of Systematic Reviews (PROSPERO) on 3 March 2022, with the identification number CRD42022314453. The study followed the updated PRISMA 2020 guideline for reporting systematic reviews and meta-analyses [19,20] to identify human biomonitoring studies that determine the prevalence and identify risk factors of genotoxic pesticide exposure among agricultural workers in Arab countries (Supplementary Table S1). The study was conducted between 1 April and 28 June 2023. Observational studies (cross-sectional, case–control, and cohort studies) were systematically searched in PubMed (NLM), Scopus (Elsevier), Web of Science—Core Collection (Clarivate), Embase, Agricola (EBSCOhost), and Index Medicus for the Eastern Mediterranean IMEMR (WHO) on 6 May 2023, based on the predefined PECO statement (Table 1). The search was conducted without any limitations or filters except for language (English, Arabic, and French). The search strategy, designed in collaboration with a medical librarian (LÖ) utilizing PubMed’s MeSH (Medical Subject Headings), involved employing a combination of four domains of search terms. These domains encompassed pesticides, agricultural workers, Arab countries, and genotoxicity outcomes as well as any diseases potentially associated with pesticide effect. The complete search strings for all databases are available in Supplementary Table S2. Additionally, two independent reviewers manually screened the reference lists of included studies.

**Figure 1:**
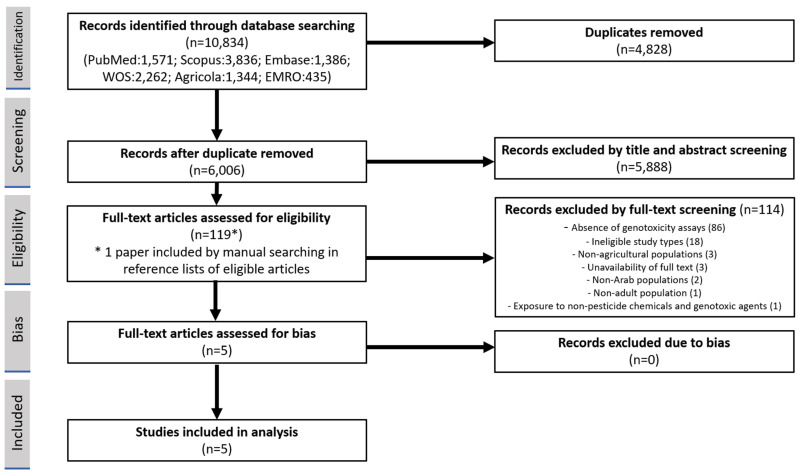
PRISMA flowchart of study selection.

The identified records of the literature search were imported to the systematic review software Covidence (Veritas Health Innovation, 2023), which was used for screening and selection. Covidence is designed to ensure blinding in all its modules [21]. Two reviewers (MSS, KRM) independently evaluated the titles and abstracts of the studies on the basis of the inclusion and exclusion criteria. If a study was deemed eligible, the full text was retrieved by the National Medical Library team at UAEU and screened by the same reviewers independently. Any discrepancies in the selection process were resolved by a third reviewer (BÁ) using the blinded conflict module in Covidence. The results of the screening process were documented using the PRISMA flow diagram (Figure 1), which included the reasons for full-text exclusion [20]. To ensure the academic integrity and reliability of the eligible studies published in open access journals, Cabell’s Predatory Reports, a trusted and comprehensive database of predatory publishing practices, was consulted. By utilizing this resource, the researchers were able to assess the credibility and legitimacy of the journals included in their study, safeguarding against potential risks of unreliable or deceptive publications [22].

### 2.3. Assessment of Study Eligibility

The following inclusion criteria were considered in selecting studies for this review: (1) original empirical research published in a peer-reviewed journal and written in English, Arabic, or French languages; (2) observational studies, including cross-sectional, case–control, and cohort studies, focusing on human biomonitoring to ascertain the prevalence, extent, and identifiable risk factors of genotoxic pesticide exposure among adult agricultural workers in Arab countries; and (3) assessment of genotoxicity endpoints.

The following studies were excluded from the systematic review: (1) in vitro, in silico, or animal studies, case reports, opinion articles, commentaries, letters, review articles, clinical trials, published abstracts, and conference proceedings; (2) studies that only evaluated the pesticidal activity of formulation(s) without examining their unintended adverse effects; (3) studies that failed to report genotoxicity endpoints or only reported cytotoxicity outcomes; and (4) studies that lacked a full-text version which was not accessible through contact with the authors.

### 2.4. Risk of Bias (RoB) in Individual Studies

The tool for assessing the RoB in this study was created in Microsoft Excel (Version 2018), based on the Navigation Guide RoB tool, which was specifically designed for systematic reviews in occupational health [23] and assesses domains such as selection bias, ascertainment bias, accuracy of exposure and outcome evaluation, and selective reporting. We adopted additional domains from the RoB-SPEO tool, which evaluates biases related to studies estimating the prevalence of exposure to occupational risk factors, such as differences in numerator and denominator, other biases, and conflicts of interest. The World Health Organization (WHO) and the International Labour Organization (ILO) jointly developed this tool for the estimation of work-related burden of disease and injury (WHO/ILO Joint Estimates) [24].

To ensure that the RoB tool was effective, it was pilot-tested on two articles. Two reviewers (MSS, KRM) independently evaluated the RoB of the selected studies across 10 distinct domains as low (dark green), probably low (light green), probably high (light red), and high (dark red). In case of any discrepancies between the judgements of the two reviewers, a third reviewer (BÁ) made the final decision (Table 2). Finally, a collective judgement was made on the eligibility of the studies based on the extent of risk of bias.

### 2.5. Data Extraction

The data extraction process was conducted by two separate reviewers (MSS, KRM) using Microsoft Excel-based data extraction sheets. The extraction sheets were developed specifically for this study and underwent pilot testing. The information extracted for this study included data on the publication (title, DOI, year of publication, first author’s name), the settings of the studies (country, study type, period of data collection), the study population (baseline characteristics of both exposed and non-exposed participants), the exposure (type, extent, and pattern of pesticide exposure), the outcome (genotoxicity tests applied, measured endpoint(s) of genetic damage, prevalence, average level and dispersion of measured DNA damage, comparison of results in exposed and non-exposed populations), cytotoxicity and health effects, contributing and risk factors, and applied preventive measures. Information on conflicts of interest, ethics, and funding was also collected.

### 2.6. Data Synthesis

We analyzed the available data from the eligible studies using a narrative approach. The extracted data are presented in a summary table (Table 3) and descriptively discussed.

**Figure 2:**
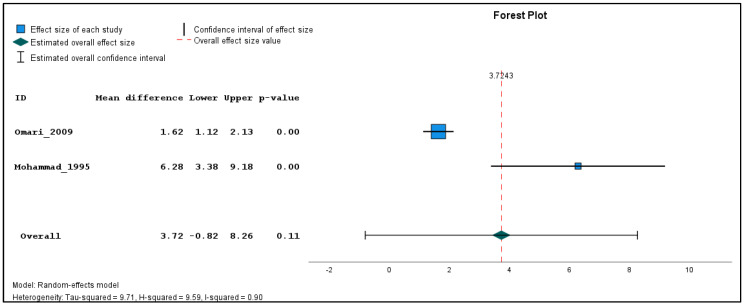
Pooled mean difference in the aberrations per 100 cells in the exposed compared to unexposed individuals, regardless of the season.

**Figure 3:**
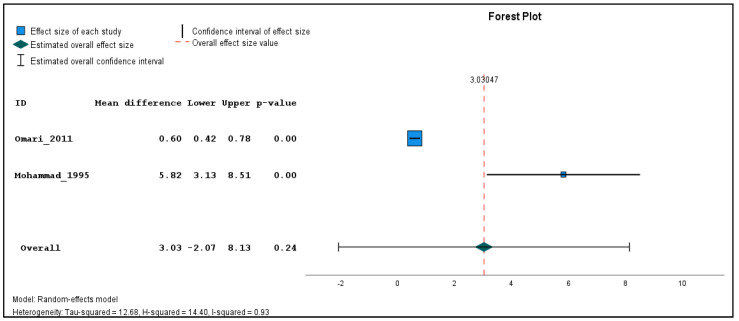
Pooled mean difference in the chromatid breaks per 100 cells in the exposed compared to un-exposed individuals, regardless of the season.

**Figure 4:**
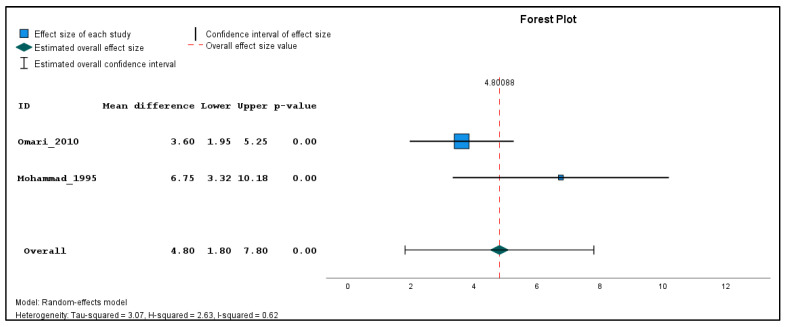
Pooled mean difference in the breaks per 100 cells in the exposed compared to un-exposed individuals, regardless of the season.

In addition, two studies were found clinically sufficiently homogenous to pool effect estimates in a quantitative meta-analysis. The inverse variance method with a random-effects model was used to quantify the weighted unstandardized mean difference between exposed and unexposed groups for three types of outcomes (genotoxicological endpoints). If the same outcome was measured in different seasons, a single mean and standard deviation value was produced using the reported individual-based data before carrying out the meta-analysis for each outcome across the different time points. The meta-analysis was performed using the Statistical Package for the Social Sciences (SPSS) software (version 29). The combined estimations are illustrated in forest plots (Figure 2, Figure 3 and Figure 4). Because only two studies were included in the meta-analysis, subgroup and sensitivity analyses could not be performed.

## 3. Results

### 3.1. Identification of Eligible Studies

The search and screening processes are presented in a PRISMA flow diagram (Figure 1). Initially, 10,834 studies were identified through the database search, and, after removing duplicates and two rounds of screening, 4 studies met the inclusion criteria. In full-text screening, 114 studies were excluded out of the 118 assessed: 86 due to the absence of genotoxicity assays, 18 for ineligible study types, 3 for non-agricultural populations, 3 due to unavailability of full text, 2 for non-Arab populations, 1 for non-adult population, and 1 for studying exposure to non-pesticide chemicals and genotoxic agents. Additionally, one record was identified by manually searching the reference lists. All eligible publications could be retrieved online. All studies included in the analysis were deemed eligible and none were excluded based on the assessment of risk of bias, as indicated in Table 2.

### 3.2. Summary of Results of Included Studies

The primary outcomes of the included studies are discussed narratively and illustrated in a summary table (Table 3). The results of two included studies were pooled for meta-analysis on the effect size of genotoxic occupational pesticide exposures in Arab countries.

Two out of the five studies that met the inclusion criteria were conducted in Egypt [25] and Syria [26], respectively. The other three studies were carried out in Jordan [27,28,29], two of which were conducted by the same laboratory [27,28]. None of the remaining 16 countries produced any studies on this topic. All the five studies were cross-sectional with no specific start or end dates reported, except for Mohammad 1995 [26], which was conducted between April and October 1994.

Amr 1999 [25] conducted a cross-sectional study in Egypt involving 300 pesticide formulators and 300 pesticide applicators. Cytogenetic changes were assessed in a subset of 32 applicators, 39 formulators, and 20 controls, with a distinct control group for both formulators and applicators. The participants were exposed to various pesticides for 5–25 years, with pesticide spraying occurring three times annually on cotton crops. The genotoxicity of participants was assessed using the chromosome aberration assay. Exposed individuals, including both formulators and applicators, exhibited significantly higher frequencies of chromosomal aberrations compared to non-exposed individuals. t-tests revealed significant differences (*p* < 0.001) in Gap, Exchange, and Dicentric, and significant differences (*p* < 0.05) in Break, Fragment, and Deletion between formulators and applicators. No additional risk factors for genotoxicity were investigated in the study. The comprehensive assessment in this study also revealed that pesticide-exposed individuals exhibited a range of health effects, including neuropsychiatric manifestations, polyneuropathy, sensory hypoesthesia, abnormal deep reflexes, psychiatric disorders (such as depressive neurosis), irritability, erectile dysfunction, liver function abnormalities, topical eye changes, gastrointestinal issues, and genitourinary manifestations.

Mohammad 1995 [26] conducted a cross-sectional study in Syria involving pesticide-exposed participants, including a sprayer group (n = 9) and a dealer and quality controller group (n = 7), compared to a non-exposed control group (n = 6). The sprayer group was sampled at the beginning, in the middle, and at the end of the spraying season. They investigated genotoxicity using the chromosome aberration assay. The study found that chromatid breaks were the most common type of structural chromosome aberrations, while dicentric chromosomes, rings, and double minutes were rare. The control group exhibited a lower percentage of chromatid breaks at 4.4% ± 1.39%. The dealers and quality controller group exhibited a significantly higher percentage of chromatid breaks with an average of 12.14% ± 3.84% (*p* < 0.05) when compared to the control group, with significantly more frequent overall genetic damage: a frequency of 37.6 (*p* < 0.05). Similarly, the sprayer group displayed significant differences in chromosomal aberrations at different stages of the season in comparison to the control group. At the beginning of the season, the percentage of chromatid breaks was 4.5 (*p* < 0.05), which increased to 26 (*p* < 0.05) in the middle of the season and further to 45 (*p* < 0.05) towards the end of the season. No additional risk factors for genotoxicity were investigated in this paper.

Omari 2009 [27] conducted a cross-sectional study in Jordan involving 40 exposed farmers and 30 non-exposed individuals to assess the genotoxic effects of pesticide exposure by chromosome aberration assay. The exposed participants, who used insecticides such as malathion and chlorpyrifos for 2 to 5 years, showed significantly elevated rates of abnormal cells, gaps, chromatid breaks, and chromosomal aberrations compared to the pesticide-non-exposed control group among both smokers and non-smokers (*p* < 0.05 for stratified analysis, *p* < 0.01 for combined analysis). In both exposed and non-exposed groups, smokers had a higher average of abnormal cells and a higher rate of aberrations per 100 cells compared to non-smokers. The same authors, Omari [28], conducted a cross-sectional in Jordan in 2011 involving 23 exposed farmers and 22 non-exposed individuals. The exposed participants, who used an insecticide mixture of malathion and chlorpyrifos for 3 to 30 years and were exposed in the last 8 months, exhibited a significant increase in DNA damage as indicated by micronucleus formation in binucleated lymphocytes (*p* < 0.01). Even eight months after exposure, the exposed group still showed a significant increase in DNA damage compared to the non-exposed group (*p* < 0.05). The mitotic index was significantly decreased in the exposed group. No specific additional risk factors were identified.

Qaqish 2016 [29] conducted a cross-sectional study to investigate the genotoxic effects of pesticide exposure among farmers in Jordan. The study sample consisted of 96 exposed participants and 96 non-exposed individuals. Pesticide exposure was assessed by the frequency of the chromosomal translocation BCL2-IGH t(14;18), using a nested polymerase chain reaction (PCR) assay targeting the major breakpoint region (MBR) of the BCL2-IGH biomarker. The results revealed a significant association between pesticide exposure and an elevated frequency of the BCL2-IGH t(14;18) translocation in farmers compared to the control group (*p* < 0.0001; odds ratio (OR) = 13.5; 95% confidence interval (CI) = 6.3–28.6). Notably, the study identified the use of insecticides on animals and pesticide application on open field crops as significant contributing factors for genetic damage.

The Investigated subjects in all studies were only males. The age ranges and mean ages of the exposed and unexposed populations varied in the five studies. The exposed populations were composed of sprayers/applicators, dealers and quality controllers, and formulators, with a variety of age ranges from 19 to 62 years. The unexposed control groups had a wider age range from 23 to 67 years, with mean ages ranging from 26.1 to 36.1 years. The total average age of the exposed population was 29.15 years, while the unexposed control group had an average age of 30.06 years.

The studies analyzed in this review investigated the toxicity of frequently used pesticides. However, none of them provided information on the active and inert components, or the concentration of the pesticides, with the exception of that of Mohammad 1995 [26], which reported the vehicle and concentration of the pesticides used for the sprayer group (150 L of deltamethrin 0.1 g/L in water and cypermethrin 1.3% in diesel per day). Furthermore, none of the studies commented on the authorizations of the pesticide formulations, chemical abstracts service (CAS) registry numbers, exposure settings (direct or indirect), route (inhalation, skin contact, ingestion), and level of exposure.

The specific types of pesticides used varied widely across the studies. While some articles provided specific brand names and chemical compositions of the pesticides used, others did not, which could limit the generalizability of their findings. For example, Mohammad 1995 [26] examined the effects of deltamethrin and cypermethrin on a sprayer group and a dealer and quality controller group, respectively. The sprayer group was exposed to Kothrine flow 25 (deltamethrin) and cymperator (cypermethrin), while the dealer and quality controller group were exposed to a mixture of pesticides available in the Syrian market, including pyrethrins. On the other hand, Omari 2011 [28] did not mention the specific types of insecticides used. These variations also extended to the purpose of use and the form of application. For example, the study by Qaqish [29] examined 96 farmers, the majority of which (80.2%) applied pesticides on open field, 47.9% used insecticides on animals, and almost all (95.8%) used herbicides.

The duration and time pattern of exposure to pesticides In these five articles varied greatly. The differences in duration of exposure between the articles reflect the diversity of pesticide-related occupations and practices, with some workers exposed for only a few years [26,27] and others for up to several decades [25,28,29]. The time pattern of exposure also varied, with some workers experiencing seasonal exposure [25,27], while others were exposed continuously throughout the year [26]. Some articles did not report the time pattern of exposure [28,29], which limits the interpretation of their findings.

All the studies analyzed blood samples to evaluate the genotoxicity of pesticides. The number of samples/individuals varied among the studies [25,27,29], with two studies collecting samples at multiple time points [26,28]. The time of sample collection varied among the studies, with Mohammad 1995 [26] collecting samples during the summer season starting mid-April and throughout the spraying season ending in October, while others [25,27,28,29] did not report the time of sample collection. Samples were collected at three-month intervals in the study from Mohammad 1995 [26], while the study by Omari 2011 [28] collected samples at an eight-month interval for both exposed and non-exposed participants. The interval of sample collection could allow for the detection of potential changes in genotoxicity over time.

The studies used blood samples for genotoxicity testing. The chromosome aberration assay was the most commonly used test to evaluate genotoxicity of pesticides [25,26,27] that involve the evaluation of chromatid breaks, chromatid exchanges, chromosomal breaks, dicentrics, and rings. Omari 2011 [28] used the micronucleus test to evaluate micronuclei frequency within the cells, while nested PCR assay was applied by Qaqish 2016 [29] to specifically target the particular biomarker of BCL2-IGH t(14;18) fusion, which is one of the most common chromosomal abnormalities in non-Hodgkin’s lymphoma (NHL).

The level of detectable DNA damage was higher in exposed individuals compared to non-exposed individuals. For example, Qaqish 2016 [29] found that farmers occupationally exposed to pesticides were 13.5 times more likely to carry the chromosomal translocation t(14;18) )63.5% of farmers compared to 11.5% of controls) [29]. Amr 1999 [25] examined the level of chromosomal aberrations in formulators (n = 39) and applicators (n = 32) exposed to pesticides, and found that they had two times higher chromosomal aberrations than the controls (n = 20), *p* < 0.001. Another investigation in the same study comparing the exposed group (n = 100) and the control group (n = 25) demonstrated significant disparities in chromosomal aberrations. The exposed group exhibited higher mean values of breaks (0.33) and gaps per cell (0.20) compared to the control group (0.039 and 0.02, respectively), with all differences being statistically significant (*p* < 0.001). Moreover, the combined measure of breaks and gaps per cell was notably higher in the exposed group (0.51) compared to the control group (0.02).

The results showed that the level of DNA damage in some studies increased with duration of exposure, while in others it did not. For example, Mohammad 1995 [26] examined the level of chromosome aberrations in sprayers, and in dealers and quality controllers exposed to pesticides. In the sprayer group, there was a gradual increase in the number of abnormal metaphases from the beginning (7.13 ± 1.4%) to the middle (9.3 ± 1.4%), and the end of the spraying season (13.3 ± 2.7%), with a significant difference in breaks between the control group and the sprayer group at each stage of the season (*p* < 0.05). The findings indicate an association between pesticide exposure time and increased chromosome aberrations in the sprayer group. Likewise, Omari 2011 [28] examined micronuclei frequency in exposed and non-exposed groups using lymphocyte analysis. Following 8 months of exposure, the exposed group demonstrated a highly significant increase in micronuclei frequency compared to the non-exposed group (*p* < 0.01). The examination of 11,500 binucleated lymphocytes revealed that 201 cells had one micronucleus (MN), 28 cells had two MNs, 26 cells had three MNs, and 15 cells had four MNs. Furthermore, even after discontinuing exposure for 8 months, the exposed group still exhibited a significant increase in micronuclei frequency compared to the non-exposed group (*p* < 0.05). Analysis of 11,500 binucleated lymphocytes from this group revealed 128 cells with one MN, 19 cells with two MNs, 6 cells with three MNs, and 2 cells with four MNs. In contrary, there were no significant associations in Qaqish 2016 [29] between BCL2-IGH t(14;18) fusion frequency and the mean duration of pesticide use (BCL2-IGH t(14:18) positive 10.6 ± 7.9 years vs. negative 11.7 ± 8.2 years, *p* = 0.51).

Furthermore, some studies found that the type of job, such as being a formulator or an applicator, could affect the level of DNA damage. In the study by Amr 1999 [25], the damage in applicators was more significantly elevated than among formulators, compared to the control. Formulators demonstrated a lower number of gaps, breaks, and deletions.

A limited number of articles have incorporated confounding factors in their analysis to prevent biased interpretations. Qaqish 2016 [29] observed younger age and higher alcohol consumption in the exposed group compared to the unexposed control group (*p* = 0.003 and 0.023, respectively). However, the study indicated that the mean age and alcohol intake were not significantly different between t(14;18)-positive and -negative cases. In Amr 1999 [25], controls were selected to have the same educational level and socio-economic status as the study group to mitigate confounding factors. In contrast, Omari 2009 [27] addressed confounding by stratifying the sample according to smoking status and excluding individuals exposed to agents that could interfere with the results, but no further adjustment was made for potential confounders, such as age and duration of exposure. Qaqish et al. 2016 [29] investigated a wide range of factors potentially contributing to BCL2-IGH t(14;18) fusions in farmers. Through the adjustment of confounding variables, including age, sunlight exposure, alcohol intake, smoking, and use of personal protective equipment, the authors revealed that the risk of BCL2-IGH t(14;18) fusion is significantly associated with pesticide exposure on open-field crops and insecticide use on animals, but not with the aforementioned confounding factors (OR = 3.0, 95% CI = 1.1–8.5, *p* = 0.03; OR = 2.4, 95% CI = 1.02–5.7, *p* = 0.043, respectively).

Furthermore, smoking was studied in two papers [27,29] as a risk factor. However, findings were conflicting, probably because of the different biomarkers. Omari 2009 [27] found an increase in the level of chromosomal aberrations in exposed compared to non-exposed individuals, and this finding remained when stratifying for smoking. On the other hand, the level of abnormality was higher in smokers than in non-smokers in both groups. Specifically, the total number of aberrations per 100 cells in pesticide-non-exposed smokers was 4.59 ± 0.35, while in pesticide-non-exposed non-smokers it was 2.04 ± 0.21. Similarly, the same values in pesticide-exposed smokers and non-smokers were 6.10 ± 0.23 and 5.13 ± 0.28, respectively. In contrary, Qaqish 2016 [29] found no significant difference in the proportion of smokers among t(14;18) positive and negative cases (66.7% vs. 65.8%, *p* = 0.9).

Only two of the five studies investigated cytotoxicity in individuals exposed to pesticides. These studies utilized different indirect biomarkers to detect cytotoxicity, such as mitotic index [28] or comprehensive measurements of hematological and biochemical parameters [25]. Omari 2011 [28] found that individuals exposed to pesticides had significantly lower mitotic index (*p* < 0.05). In contrast, Amr 1999 [25] found no significance in all measured parameters, such as absolute eosinophils, absolute polymorph nuclear cells, absolute lymphocytes, absolute monocytes, and phagocytic index, that could potentially provide insights into immune system responses and inflammation, which are indirectly relevant to cytotoxicity.

Meta-analysis was performed on two studies, namely Mohammad 1995 [26] and Omari 2009 [27], which utilized the same genotoxicity assay (chromosome aberration assay) and reported the result in a comparable way. Although Amr 1999 [25] also detected chromosomal aberrations, it had to be excluded from the meta-analysis due to lack of comprehensive description of the analysis, types of breaks assessed, and the unclear explanation for using only a subgroup of the study population for cytogenetic studies, which is reflected in the high risk of bias due to selective reporting of exposures/outcomes (Table 2). Both included studies assessed chromatid breaks, chromosome breaks, chromatid exchanges, dicentrics, and rings. However, Mohammad 1995 [26] additionally included double minutes (DMs), while Omari 2009 [27] included trivalents and gaps, whose outcomes were excluded from the meta-analysis for coherence. To ensure consistency, the means and standard deviations of the total number of aberrations/100 cells, weighted sum of breaks/100 cells, and number of chromatid breaks/100 cells were recalculated from the reported individual raw data. In the weighted sum, a chromatid break was considered as one, a chromosome break and a chromatid exchange as two, and dicentrics and rings as four breaks. The exposed population in the study by Mohammad 1995 [26] was assessed at three different time points during the spraying season, the data of which were combined to a single mean and standard deviation for the meta-analysis.

The results of the meta-analysis revealed that while there were no statistically significant differences in the pooled frequency of chromosomal aberrations (Figure 2) between the exposed and unexposed groups, there was a moderate increase observed (mean difference = 3.72, 95% CI: −0.82 to 8.26, *p* = 0.11). Similarly, the pooled analysis on chromatid breaks frequency (Figure 3) did not yield a statistically significant result (mean difference = 3.03, 95% CI: −2.07 to 8.13, *p* = 0.24). However, the pooled analysis demonstrated a significant elevation in the weighted sum of breaks (mean difference = 4.80, 95% CI: 1.80 to 7.80, *p* < 0.001) among the exposed individuals (Figure 4). These findings suggest a potential association between pesticide exposure and increased chromosomal aberrations measured by the weighted sum of breaks, even though the specific types of chromosomal aberrations did not show statistical significance.

Furthermore, the meta-analysis revealed significant heterogeneity in the two studies examining the frequency of chromosomal aberrations, sum of breaks, and chromatid breaks, with tau-squared values of 9.71, 3.07, and 12.68, and H-squared values of 0.90, 0.62, and 14.40, respectively, indicating substantial variation beyond standard error. These significant heterogeneity values suggest that factors other than random variation may contribute to the observed differences, such as variations in study design, participant characteristics, pesticide types, and exposure levels.

## 4. Discussion

Farmworkers in developing countries often live in poor conditions and receive inadequate field training to understand the regulations that aim to minimize their pesticide exposure. Estimates suggest that a significant percentage of workers in developing countries are either unaware of PPE or do not use it, and the majority frequently misunderstand the pictograms on pesticide labels [30,31,32,33]. In addition to application, pesticide storage and disposal are activities with significant risk of exposure not only at work but also for the general population, as inappropriate storage and disposal can lead to pesticide contamination of food and water sources [30].

This systematic review presents a comprehensive analysis of the limited literature available on the association between pesticide exposure and genotoxicological effect in Arab countries, particularly focusing on the high-risk population of agricultural workers.

We observed a limited representation of research conducted in Arab countries focusing on the assessment of DNA damage in agricultural workers exposed to pesticides. Specifically, only five eligible studies originating from 3 Arab countries were eligible for analysis (15% of countries in the MENA region), and studies addressing this topic were identified in the remaining 16 countries. This scarcity of research in the broader Arab region highlights a significant gap in our understanding of the genotoxic effects of pesticide exposure in the agricultural context.

The findings of this systematic review reveal a crucial positive association between pesticide exposure and DNA damage in Arab countries, which is not only supported by the individual study findings, but also by the pooled result of the meta-analysis on the weighted sum of breaks. These findings are consistent with the previous literature from other regions of the world [34,35,36], emphasizing the potential health risks associated with pesticide exposure in agricultural settings. However, it is important to note that the scarcity of research in the broader Arab region indicates a significant gap in our understanding of this issue locally.

The eligible studies employed various methods, such as assessing structural aberrations in metaphase chromosomes, analyzing micronuclei in binucleated lymphocytes, and detecting chromosomal translocations to evaluate DNA damage. The limited availability of suitable homogenous studies highlights the need for more comprehensive and standardized biomonitoring studies to assess the genotoxic effects of pesticide exposure in agricultural workers in the Arab region.

The studies included in the review examined the genotoxicity of commonly used pesticides but failed to disclose the composition of formulations. None of the studies commented on their authorization, CAS registry numbers, exposure settings (direct or indirect), and routes (inhalation, skin contact, ingestion). The lack of specificity in most of the articles regarding the types and ingredients of pesticides used, and the potential variations in the duration, time pattern, and exposure levels could limit the generalizability of their findings. These shortcomings of exposure assessment limit the specificity of the detected genotoxic effect. The discrepancies in pesticide use, even within the same geographical location, can be attributed to factors such as target pests, crop types, associated pests or diseases, and farming practices like crop rotation and integrated pest management techniques [37]. Hence, it is crucial to conduct more comprehensive investigations into the toxicity of commonly used pesticides, taking into account the specific types of pesticides and variation in exposure patterns, which are essential for gaining insights into the risk of developing health problems associated with pesticide exposure. In addition, it is crucial to consider the simultaneous exposure to multiple pesticides, as their combined genotoxic effect can be synergistic [38]. For instance, Amr 1999 [25] examined the exposure to chlorinated hydrocarbons, organophosphates (dimethoate, malathion, dichlorvos), carbamates (propoxur), and pyrethroids (cypermethrin, deltamethrin, tetramethrin, sumithrin, D-allethrin). In contrast, Omari 2009 [27] specifically investigated the effects of malathion and chlorpyrifos [20].

Only two studies [27,29] investigated the effects of smoking as a contributing factor in individuals exposed to pesticides, in spite of its well-known strong genotoxicity [39], which was clearly demonstrated by Omari 2009 [27].

When cells encounter toxic substances like pesticides, their vitality and functionality may be compromised, initiating cellular damage that can subsequently result in genetic modifications and DNA damage [40]. This critical information aids in understanding the mechanisms through which pesticides induce genotoxic effects either directly or indirectly through decreased cell viability. Only two studies [25,28] in this review investigated cytotoxicity, using indirect biomarkers. These studies employed different methods to detect cytotoxicity and reported conflicting results.

The comprehensive assessment by Amr 1999 [25] revealed that pesticide-exposed individuals exhibited a range of health effects. The observations on symptoms and diseases among pesticide-exposed agricultural workers suggest that pesticide exposure can pose significant harm to various organ systems and may result in a broad spectrum of health effects in addition to DNA damage and its consequences, as described in several publications [34,41]. While four of the included studies did not provide specific prevalence rates of symptoms or diseases, it is important to note that the presence of genotoxic consequences, such as DNA damage, still allows for conclusions to be drawn regarding the effectiveness of preventive measures in mitigating these effects.

The number of exposed and unexposed individuals varied greatly among the studies. For instance, Amr 1999 [25] had the highest number of exposed participants at 600 (300 pesticide formulators and 300 pesticide applicators), along with 400 non-exposed participants. In contrast, Mohammad 1995 [26] had the lowest number of exposed participants at 16 (9 in the sprayer group and 7 in the dealer and quality controller group), and only 6 non-exposed participants. Another important limitation was the presence of uncontrolled confounding factors, particularly when occupational workers were exposed to various types of pesticides and other genotoxic agents. It is noteworthy that two of the studies did not adequately control for potential confounding factors, while all the five studies solely focused on male participants. These limitations in study design and participant selection may introduce bias into the results, e.g., gender bias. Data indicate that globally women make up 43% of the agricultural labor force, with a greater proportion employed in agriculture in developing countries in South Asia and the Middle East compared to men [42]. In Northern Africa, there has been a notable increase in the proportion of women employed in agriculture from 30% to almost 45% [42].

The majority of the articles did not address the occupation or potential exposures of the unexposed control population. This is noteworthy as three of the articles had controls who lived in the same residential areas as the exposed population, while only one study [29] stated explicitly that the controls had no history of farming-related work. The available literature indicates that people living near agricultural areas may be at risk of exposure to pesticides through non-occupational pathways, such as the drift and volatilization of pesticides beyond the treated area [43]. The inadequate knowledge and use of proper safety measures in handling pesticides in Arab countries have been reported and can eventually lead to potential exposure of the general population, including those in the control groups of the reviewed articles. For instance, in a study conducted in Kuwait [44], over 70% of farmers did not adhere to the instructions on pesticide labels, and 58% did not utilize personal protective equipment while handling pesticides. Such poor knowledge contributes to increase in the pesticide residues in food. In the UAE, 4513 samples of fresh fruits were tested between 2018 and 2020, and 81 different pesticide residues were detected. In 73.2% of the samples, pesticide levels exceeded the maximum residue limit (MRL) [45].

### Strengths and Limitations

The systematic review presented here exhibits several notable strengths. It provides the first comprehensive examination of the existing literature concerning the relationship between pesticide exposure and genotoxicological outcomes in Arab countries, particularly focusing on the high-risk population of agricultural workers. A comprehensive and clear research question was outlined in a PECO statement, which provided a structured and focused approach to the review. The study protocol has been registered in the PROSPERO, and the review adheres to the PRISMA-P statement. We have adopted comprehensive and transparent search strategy, and multiple databases have been searched. The use of Covidence software ensured blinding, consequently reducing the risk of reviewer bias. The RoB tool created for this study is specific to occupational health, which is appropriate for the research question. By systematically reviewing multiple studies, this review offers a more comprehensive understanding of the subject matter by consolidating the available evidence. It concentrates specifically on agricultural workers, a population at high risk, allowing for the identification and elucidation of potential risks associated with increased pesticide exposure during farming activities. Additionally, the review recognizes the significance of confounding factors and provides a critical analysis of how different studies have addressed them. By acknowledging the role of confounders and discussing their inclusion or exclusion in the analyzed studies, the review offers a nuanced perspective on the impact of these factors on the reported associations.

It is crucial to distinguish between the methodological limitations of the systematic review and the limitations inherent in the findings themselves. The methodological limitations, such as the language bias resulting from the search being limited to English, Arabic, and French, are acknowledged. However, these limitations do not compromise the overall integrity of the review. On the other hand, the limitations of the findings primarily stem from the scarcity of high-quality publications and data regarding pesticide exposure and its genotoxic effects in Arab countries, as discussed above. These limitations restrict the scope of the conclusions that can be drawn from the studies’ synthesized results.

The meta-analysis conducted in this study has several limitations that should be considered. Firstly, the analysis was based on only two studies, Mohammad 1995 [26] and Omari 2009 [27]; the study by Amr 1999 [25] had to be excluded from the meta-analysis due to its directly non-comparable and poorly reported results. The limited number of studies did not allow for conducting subgroup and sensitivity analysis. Secondly, while both studies utilized the chromosome aberration assay to assess similar parameters, there were some differences in the specific measurements performed and therefore the identical parameters (number of aberrations, weighted sum of breaks, and number of chromatid breaks per 100 cells) had to be recalculated from the raw data. Another limitation arises from the differences in the timing of data collection between the two studies. Mohammad 1995 [26] measured the exposed population at three different time points during the spraying season, while Omari 2009 [27] relied on a single time point measurement without specific information on the timing. Consequently, the results in the three time points had to be pooled for meta-analysis. Caution should be exercised when interpreting the combined risk estimate due to the potential heterogeneity introduced by the different modes of action of the pesticides used in the included studies. Despite these limitations, the meta-analysis provides plausible findings that are in line with the literature.

## 5. Conclusions

This systematic review provides a comprehensive analysis of the existing literature on the genotoxicity of pesticide exposures in agricultural workers of Arab countries. The findings highlight the limited number of studies available, emphasizing the need for further research in this area.

The discovered methodological limitations of the reviewed studies underpin the need for more comprehensive investigations with larger sample sizes, more precisely selected controls, detailed exposure assessment, utilization of cytotoxicity assays and adjustment for potential confounders to enhance our understanding of the genotoxicological risks linked to pesticide exposure. To enhance the validity and reliability of genotoxicity measurements, future studies should provide comprehensive information on laboratory protocols, including control samples.

The synthesized findings provide valuable information not only to the scientific community but also to local policymakers. By addressing the identified gaps and limitations in future studies, we can gain a better understanding of the potential health risks associated with pesticide exposure and develop appropriate preventive strategies to protect the health and well-being of individuals in this region.

## Figures and Tables

**Table 1:** PECO (population, exposure, comparator, outcome) statement.

PECO Element	Description
Population	Adult (>18 years old) professional agricultural workers, defined as farmers and pesticide applicators, in Arabic-speaking countries of the MENA region (19 countries: Algeria, Bahrain, Egypt, Iraq, Jordan, Kuwait, Lebanon, Libya, Morocco, Mauritania, Oman, Palestine, Qatar, Saudi Arabia, Sudan, Syria, Tunisia, the United Arab Emirates, and Yemen), while those who work in other sectors, who are located outside the region, and who are less than 18 years old were excluded.
Exposure	Exposure to a variety of pesticide products used in agricultural settings, while excluding exposure to non-agricultural pesticides, other chemicals, and genotoxic agents.
Comparator	No comparators were used for assessing the prevalence and extent of DNA damage. The comparator group for identifying and determining the effect size of genotoxic pesticide exposures and risk factors were populations not directly exposed to pesticides, or the general population.
Outcome	Biomarkers of DNA damage detected by established genotoxicity tests, such as DNA strand break measurements, cytogenetic assays, and mutagenicity assays. Additional outcomes included prevalence and risk factors of genotoxicity among agricultural workers exposed to pesticides in Arab countries.

**Table 2:** Risk of bias in the selected studies investigating genotoxicity of occupational pesticide exposure in Arab countries.

Risk of Bias Domain	1—Bias in Selection of Participants into the Study	2—Bias Due to a Lack of Blinding of Study Personnel	3—Bias Due to Exposure Misclassification	4—Bias Due to Incomplete Exposure Data	5—Bias Due to Outcome Misclassification	6—Bias Due to Selective Reporting of Exposures/Outcomes	7—Bias Due to Differences in Numerator and Denominator	8—Bias Due to Confounding	9—Bias Due to Conflicts of Interest	10—Other Bias
Amr 1999 [25]										
Mohammad 1995 [26]										
Omari 2009 [27]										
Omari 2011 [28]										
Qaqish 2016 [29]										

**Table 3:** Characteristics of the selected studies investigating genotoxicity of occupational pesticide exposure in Arab countries.

Country	Exposed Participants	Non-Exposed	Type of Pesticide, Duration, and Pattern of Exposure	Assay and Type of Biomarker	DNA Damage in Exposed Participants	DNA Damage in Non-Exposed Participants	Comparison of Exposed and Non-Exposed Participants	Additional Risk Factors/Confounders	Ref.
Egypt	300 pesticide formulators 300 pesticide applicators Cytogenetics was assessed in only 32 applicators and 39 formulators	20 to compare with applicators, another 20 to compare with formulators	Chlorinated hydrocarbons, organophosphates (dimethoate, malathion, dichlorvos), carbamates (propoxur), as well as pyrethroids (cypermethrin, deltamethrin, tetramethrin, sumithrin, D-allethrin); Formulators 5–25 yrs exposure, applicators 5–15 yrs exposure; pesticide spraying: 3 x/yr, June–Sept.	Chromosome aberration assay; gaps, breaks, exchanges, dicentrics, fragments, and deletions	Formulators: gaps: 1.58 ± 0.81, breaks: 1.13 ± 0.86, exchanges: 0.7 ± 0.7, dicentrics: 0.79 ± 0.6, fragments: 0.54 ± 0.6, deletions: 0.3 ± 0.5 Applicators: gaps: 4.13, breaks: 1.8, isobreaks: 0.28, deletions: 8.89 (N.B: The paper was not clear regarding the number of cells from which they calculated those averages and standard deviations)	Gaps: 1.05 ± 0.06, breaks: 0.7 ± 0.86, exchanges: 0.1 ± 0.3, dicentrics: 0.2 ± 0.5, fragments: 0.25 ± 0.4, deletions: 0.1 ± 0.3	Significant differences (*p* < 0.001) in gap, exchange, and dicentric Significant differences (*p* < 0.05) in break, fragment, and deletion between formulators and applicators	There were no additional risk factors reported	Amr 1999 [25]
Syria	9 sprayers, 7 dealers, and quality controllers	6	Sprayers: deltamethrin and cypermethrin, 3 years exposure Dealers and quality controllers: mixture of pesticides including pyrethrins; year-round exposure	Chromosome aberration assay; chromatid breaks, chromatid exchanges, chromosomal breaks, dicentrics, rings, minutes	Average number ± SD of aberrations per 100 cells in sprayers: Beginning of season: aberrations: 7 ± 1.85, breaks: 7.5 ± 2.62, chromatid breaks: 6 ± 2.69 Middle of season: 10 ± 1.32, 12.11 ± 2.37, 8.78 ± 1.72 End of season: 13.78 ± 2.73, 15.33 ± 3.43, 12.44 ± 2.65 Average number ± SD of aberrations per 100 cells in dealers and quality controllers: 13.52 ± 3.40, 15.38 ± 3.18, 11.95 ± 3.85	Aberrations: 4.34 ± 1.39, breaks: 5.16 ± 1.59, chromatid breaks: 3.64 ± 1.47	Sprayers: Significant differences in chromatid breaks at the beginning, middle and end of season (*p* < 0/05) Dealers and quality controllers: Significant difference in chromatid breaks (*p* < 0.05) and in all genetic damage (*p* < 0.05)	There were no additional risk factors reported	Moham-mad 1995 [26]
Jordan	40 farmers	30	Malathion and chlorpyrifos; Duration of exposure: 2 to 5 years	Chromosome aberration assay; gaps, chromatid breaks, isochromatid breaks, and exchanges such as dicentric, rings, and trivalents	Smokers had 5.75 ± 0.05 abnormal cells, and 6.10 ± 0.23 aberrations/100 cells, while non-smokers had 3.35 ± 0.26 abnormal cells, and 5.13 ± 0.28 aberrations/100 cells.	Smokers had 5.13 ± 0.36 abnormal cells, and 4.59 ± 0.35 aberrations/100 cells, while non-smokers had 4.14 ± 0.32 abnormal cells, and 2.04 ± 0.21 aberrations/100 cells	In both the smokers and non-smokers subsets, the pesticide-exposed group exhibited significantly higher rates (*p* < 0.05 for individual analysis, *p* < 0.01 for combined analysis) of abnormal cells, gaps, chromatid breaks, and chromosomal aberrations compared to the pesticide non-exposed control group.	Confounders such as age and duration of exposure were controlled, and the individuals were stratified based on smoking status. Significantly higher incidence of DNA damage was observed in smokers among the exposed group compared to both non-smokers within the same group and the unexposed controls (*p* < 0.05) Individuals who had been exposed to potentially genotoxic agents were excluded from the analysis	Omari 2009 [27]
Jordan	23 farmers	22	Insecticide mixture Malathion and chlorpyrifosDuration of use: 3–30 years	Micronucleus test; frequency of micronuclei (MN)	The examination of 11,500 binucleated lymphocytes revealed after 8 months of exposure: 0 MN: 11,230, 1 MN: 201, 2 MN: 28, 3 MN: 26, 4 MN: 15 cells After 8 months free from exposure: 0 MN: 11,345, 1 MN: 128, 2 MN: 19, 3 MN: 6, 4 MN: 2 cells	The examination of 11,500 binucleated lymphocytes revealed 0 MN: 10,918, 1 MN: 75, 2 MN: 7 cells, with no cells observed with 3 MN or 4 MN	After 8 months of exposure: highly significant increase in MN frequency (*p* < 0.01) After 8 months free from exposure: significant increase in MN frequency (*p* < 0.05)	There were no additional factors reported Significant decrease in mitotic index in exposed groups compared to control group; no specific causes mentioned	Omari 2011 [28]
Jordan	96 farmers	96 community members	Pesticide types not reported Open field pesticide use: 80.2% Herbicide use: 95.8% Insecticide use on animals: 47.9% Duration of exposure: 1–40 years (mean 10.9 ± 7.9 years)	Nested polymerase chain reaction (PCR) assay; BCL2-IGH t(14;18) fusion frequency	63.5% (61 out of 96)	11.5% (11 out of 96)	Significant increase for all exposure; OR = 13.5 (95%CI = 6.3–28.6), *p* < 0.0001 Significant increase for pesticide use on open fields; OR = 3.0 (95%CI = 1.1–8.5), *p* = 0.03 Significant increase for insecticide use on animals; OR = 2.4 (95% CI = 1.02–5.7), *p* = 0.043 No significant association for herbicide use; OR = 0.57 (95%CI = 0.06–5.7), *p* = 0.627	No significant association for duration of pesticide use; *p* = 0.51 No significant association for wearing a mask; OR = 0.7 (95%CI = 0.04–0.7), *p* = 0.99 No significant association for wearing a mask and gloves, OR = 2.3 (95%CI = 0.8–6.6), *p* = 0.15)	Qaqish 2016 [29]

## Data Availability

Not applicable.

## Supplementary Materials

The following supporting information can be downloaded at: https://www.mdpi.com/article/10.3390/toxics11080663/s1, Table S1: PRISMA Checklist; Table S2: Search string. This content has been supplied by the author(s). Any opinions or recommendations discussed are solely those of the author(s) and are not endorsed by *Toxics*.
