# Supplementary material for: Genotoxicity of Occupational Pesticide Exposures among Agricultural Workers in Arab Countries: A Systematic Review and Meta-Analysis
Source: Toxics. 2023 Aug 1;11(8):663. doi: 10.3390/toxics11080663 (PMC10458041; doi:10.3390/toxics11080663)
Supplement: Supplementary file 1 [file toxics-11-00663-s001.zip › toxics-2500622-Supp Table S2 Search string_rev.pdf]

Supplementary Table S2. Literature search string

Source and search date	Search string	Results and notes
**PubMed** (NLM)<br><br>**Coverage:** from database inception to search date<br><br>**Search date:** 2023-05-05	(((((farmer*[Title/Abstract] OR "farm"[Title/Abstract] OR farms[Title/Abstract] OR ranch[Title/Abstract] OR rancher*[Title/Abstract] OR agronomist*[Title/Abstract] OR smallholder*[Title/Abstract] OR grazier*[Title/Abstract] OR farmhand*[Title/Abstract] OR "Farmers"[MeSH] OR agricultur*[Title/Abstract] OR grower*[Title/Abstract] OR reaper*[Title/Abstract] OR breeder*[Title/Abstract] OR cropper*[Title/Abstract] OR cultivator*[Title/Abstract] OR feeder*[Title/Abstract] OR gardener*[Title/Abstract] OR gleaner*[Title/Abstract] OR harvester*[Title/Abstract] OR horticulturist*[Title/Abstract] OR planter*[Title/Abstract] OR tiller*[Title/Abstract] OR rural*[Title/Abstract] OR "Rural Population"[MeSH] OR "worker*"[Title/Abstract] OR "labor*"[Title/Abstract] OR "labour*"[Title/Abstract])) AND (("Bahrain"[MeSH] OR Bahrain*[Title/Abstract] OR "Iraq"[MeSH] OR Iraq*[Title/Abstract] OR "Jordan"[MeSH] OR Jordan*[Title/Abstract] OR "Kuwait"[MeSH] OR Kuwait*[Title/Abstract] OR "Lebanon"[MeSH] OR Lebanon[Title/Abstract] OR Lebanese*[Title/Abstract] OR "Oman"[MeSH] OR Oman*[Title/Abstract] OR "Qatar"[MeSH] OR Qatar*[Title/Abstract] OR "Saudi Arabia"[MeSH] OR Saudi*[Title/Abstract] OR KSA[Title/Abstract] OR "Syria"[MeSH] OR Syria*[Title/Abstract] OR "United Arab Emirates"[MeSH] OR "United Arab Emirates"[Title/Abstract] OR UAE[Title/Abstract] OR Emirat*[Title/Abstract] OR "Yemen"[MeSH] OR Yemen*[Title/Abstract] OR "Egypt"[MeSH] OR Egypt*[Title/Abstract] OR "Sudan"[MeSH] OR "Sudan*"[Title/Abstract] OR "Mauritania"[MeSH] OR "Mauritan*"[Title/Abstract] OR Maghreb*[Title/Abstract] OR Maghrib*[Title/Abstract] OR Morocco*[Title/Abstract] OR Morocco [MeSH] OR Algeri*[Title/Abstract] OR Algeria[MeSH] OR Libya*[Title/Abstract] OR Libya[MeSH] OR Tunis*[Title/Abstract] OR Tunisia[MeSH] OR Palestin*[Title/Abstract] OR "Arab World"[MeSH] OR "Africa, Northern"[MeSH] OR "Middle East*"[Title/Abstract] OR "Middle East"[MeSH:NoExp] OR "Near East"[Title/Abstract] OR "East Mediterranean"[Title/Abstract] OR "Eastern Mediterranean"[Title/Abstract] OR Arabic[Title/Abstract] OR Arabs[Title/Abstract] OR Arab[Title/Abstract] OR MENA[Title/Abstract] OR "Arabian Peninsula"[Title/Abstract] OR "North Africa*"[Title/Abstract] OR "Northern Africa*"[Title/Abstract] OR "West Bank*"[Title/Abstract] OR "Gaza Strip"[Title/Abstract] OR Levant*[Title/Abstract] OR gulf*[Title/Abstract] OR "Arabs"[MeSH] OR "Arab World"[MeSH]))) AND (("Poisons"[Mesh:NoExp] OR poison*[Title/Abstract] OR toxic*[Title/Abstract] OR	**Results:** 1,571<br><br>**Notes:**<br>All search terms are searched in the fields: "title" and "abstract" and in the MeSH (when available).

	toxicogenetic*[Title/Abstract] OR "Toxicogenetics"[MeSH] OR genotox*[Title/Abstract] OR cytotox*[Title/Abstract] OR "Cytotoxins"[MeSH] OR antimetabolite*[Title/Abstract] OR "Antimetabolites"[MeSH] OR antispermat*[Title/Abstract] OR "Antispermatogenic Agents"[MeSH] OR cardiotox*[Title/Abstract] OR "Cardiotoxins"[MeSH] OR dermatotox*[Title/Abstract] OR dermotox*[Title/Abstract] OR "Dermotoxins"[MeSH] OR hepatotox*[Title/Abstract] OR nephrotox*[Title/Abstract] OR pneumotox*[Title/Abstract] OR immunotox*[Title/Abstract] OR "Immunotoxins"[MeSH] OR neurotox*[Title/Abstract] OR "Neurotoxins"[MeSH] OR "Toxic Actions"[MeSH] OR noxae*[Title/Abstract] OR hazard*[Title/Abstract] OR "Toxicity Tests"[MeSH] OR "Mutagenicity Tests"[MeSH] OR "Carcinogenicity Tests"[MeSH] OR pharmacogenomic*[Title/Abstract] OR "Pharmacogenomic Testing"[MeSH] OR "Comet Assay"[MeSH] OR "comet assay*"[Title/Abstract] OR "Micronucleus Tests"[MeSH] OR "micronucleus test*"[Title/Abstract] OR "chromosome aberrat*"[Title/Abstract] OR "chromosomal aberrat*"[Title/Abstract] OR "Chromosome Aberrations"[MeSH] OR denaturation*[Title/Abstract] OR "Nucleic Acid Denaturation"[MeSH] OR "fluorescent in situ hybridization*"[Title/Abstract] OR "In Situ Hybridization, Fluorescence"[MeSH] OR "Nucleic Acid Hybridization"[MeSH] OR "nucleic acid hybridization*"[Title/Abstract] OR "ames test*"[Title/Abstract] OR aneuploidy*[Title/Abstract] OR "Aneuploidy"[Mesh] OR topoisomerase*[Title/Abstract] OR "DNA Topoisomerases"[Mesh] OR "Teniposide"[Mesh] OR teniposide*[Title/Abstract] OR "Etoposide"[Mesh] OR etoposide*[Title/Abstract] OR greenscreen*[Title/Abstract] OR "γH2AX*"[Title/Abstract] OR "pH2AX*"[Title/Abstract] OR "high content screening*"[Title/Abstract] OR "pH3"[Title/Abstract] OR "cycle arrest*"[Title/Abstract] OR phospho-histon*[Title/Abstract] OR phosphohiston*[Title/Abstract] OR caspase*[Title/Abstract] OR "Caspases"[MeSH] OR "tubulin microtubule*"[Title/Abstract] OR "profiling assay*"[Title/Abstract] OR steatosis*[Title/Abstract] OR "Genetic Carrier Screening"[MeSH] OR carcinogen*[Title/Abstract] OR "Carcinogens"[MeSH] OR "Carcinogenesis"[MeSH] OR mutagen*[Title/Abstract] OR "Mutagens"[MeSH] OR "Mutagenesis"[MeSH] OR mutation*[Title/Abstract] OR "Mutation"[MeSH] OR teratogen*[Title/Abstract] OR "Teratogens"[MeSH] OR "Teratogenesis"[MeSH] OR disorder*[Title/Abstract] OR genetic*[Title/Abstract] OR "DNA"[Title/Abstract] OR "DNA"[Mesh] OR "RNA"[Title/Abstract] OR "RNA"[Mesh] OR "DNA Damage"[MeSH] OR damag*[Title/Abstract] OR insult*[Title/Abstract] OR adduct*[Title/Abstract] OR alkylation*[Title/Abstract] OR "Alkylation"[MeSH] OR alkylating[Title/Abstract] OR methylation*[Title/Abstract] OR "Methylation"[MeSH] OR oxidizing[Title/Abstract] OR oxidant*[Title/Abstract] OR "Oxidative Stress"[MeSH] OR "oxidative	

	stress*"[Title/Abstract] OR "free radical*"[Title/Abstract] OR "Free Radicals"[MeSH] OR "Cell Survival"[MeSH] OR viabilit*[Title/Abstract] OR viable*[Title/Abstract] OR necro*[Title/Abstract] OR "Necrosis"[MeSH] OR apopto*[Title/Abstract] OR "Apoptosis"[MeSH] OR "Genes, Lethal"[MeSH] OR lethal*[Title/Abstract] OR "Tissue Survival"[MeSH] OR "surviv*"[Title/Abstract] OR "adverse effect*"[Title/Abstract] OR "side effect*"[Title/Abstract] OR "Long Term Adverse Effects"[MeSH] OR harmful[Title/Abstract] OR disease*[Title/Abstract] OR "Disease"[MeSH] OR illness*[Title/Abstract] OR syndrome*[Title/Abstract] OR "Syndrome"[MeSH] OR symptom*[Title/Abstract] OR abnormal*[Title/Abstract] OR irritant*[Title/Abstract] OR "Irritants"[MeSH] OR cancer*[Title/Abstract] OR neoplas*[Title/Abstract] OR "Neoplasms"[MeSH] OR tumor*[Title/Abstract] OR tumour*[Title/Abstract] OR malignan*[Title/Abstract] OR carcinoma*[Title/Abstract] OR "Carcinoma"[MeSH] OR malformat*[Title/Abstract] OR anomal*[Title/Abstract] OR abnormal*[Title/Abstract] OR "congenital defect*"[Title/Abstract] OR "birth defect*"[Title/Abstract] OR "Congenital Abnormalities"[MeSH] OR reprotox*[Title/Abstract] OR reproduct*[Title/Abstract] OR "Reproduction"[MeSH] OR allerg*[Title/Abstract] OR "Allergy and Immunology"[MeSH] OR hypersensitiv*[Title/Abstract] OR "Hypersensitivity"[MeSH] OR histolog*[Title/Abstract] OR "Histology"[MeSH] OR endocrin*[Title/Abstract] OR "Endocrine System"[MeSH] OR "Endocrine Disruptors"[MeSH] OR neuroendocrin*[Title/Abstract] OR "neuro-endocrin*"[Title/Abstract] OR "Neurosecretory Systems"[MeSH] OR physiopath*[Title/Abstract] OR pathophysi*[Title/Abstract] OR patho*[Title/Abstract] OR "Pathology"[MeSH]))) AND ((agrochemical*[Title/Abstract] OR "chemical*"[Title/Abstract] OR agrichemical*[Title/Abstract] OR "plant protection product*"[Title/Abstract] OR pesticid*[Title/Abstract] OR biocid*[Title/Abstract] OR herbicid*[Title/Abstract] OR weedkiller*[Title/Abstract] OR "weed killer*"[Title/Abstract] OR defolian*[Title/Abstract] OR insecticid*[Title/Abstract] OR nematicid*[Title/Abstract] OR molluscicid*[Title/Abstract] OR piscicid*[Title/Abstract] OR avicid*[Title/Abstract] OR rodenticid*[Title/Abstract] OR bactericid*[Title/Abstract] OR repellent*[Title/Abstract] OR antimicrob*[Title/Abstract] OR "antiparasit*"[Title/Abstract] OR fungicid*[Title/Abstract] OR lampricid*[Title/Abstract] OR acaricid*[Title/Abstract] OR miticid*[Title/Abstract] OR "mite control*"[Title/Abstract] OR algicid*[Title/Abstract] OR algaecid*[Title/Abstract] OR chemosterilant*[Title/Abstract] OR "Agrochemicals"[MeSH:NoExp] OR "Pesticides"[MeSH] OR "Antiparasitic Agents"[MeSH] OR "Insect Repellents"[MeSH])))	

**Scopus** (Elsevier)<br><br>**Coverage:** from database inception to search date<br><br>**Search date:** 2023-05-05	(TITLE-ABS-KEY (farmer* OR "farm" OR farms OR ranch OR rancher* OR agronomist* OR smallholder* OR grazier* OR farmhand* OR "farmers" OR agricultur* OR grower* OR reaper* OR breeder* OR cropper* OR cultivator* OR feeder* OR gardener* OR gleaner* OR harvester* OR horticulturist* OR planter* OR tiller* OR rural* OR "worker*" OR "labor*" OR "labour*")) AND (TITLE-ABS-KEY ("iraq*" OR "lebanon" OR "sudan" OR "mauritania" OR "middle east*" OR "near east" OR "east mediterranean" OR "eastern mediterran*" OR arabic OR arabs OR arab OR mena OR "arabian peninsula" OR "north africa*" OR "northern africa*" OR maghreb* OR maghrib* OR morocco* OR egypt* OR jordan* OR lebanes* OR syria* OR algeri* OR libya* OR tunis* OR uae OR "united arab*" OR emirat* OR saudi* OR ksa OR qatar* OR oman* OR yemen* OR kuwait* OR bahrain* OR palestin* OR "west bank" OR "gaza strip" OR levant* OR gulf*)) AND (TITLE-ABS-KEY (poison* OR toxic* OR genotox* OR cytotox* OR antimetabolite* OR antispermatogen* OR cardiotox* OR dermatotox* OR dermotox* OR reprotox* OR hepatotox* OR nephrotox* OR pneumotox* OR immunotox* OR neurotox* OR noxae* OR hazard* OR pharmacogenomic* OR "comet assay*" OR "micronucleus*" OR "aberrat*" OR "chromosom*" OR denaturation* OR "hybridization*" OR "ames" OR aneuploid* OR topoisomeras* OR teniposid* OR etoposid* OR greenscreen* OR "γh2ax*" OR "ph2ax*" OR "high content screening*" OR "ph3" OR "cycle arrest*" OR phospho-histon* OR phosphohiston* OR caspase* OR "tubulin microtubule*" OR "profiling assay*" OR steatosis* OR carcino* OR mutagen* OR "mutation" OR teratogen* OR disorder* OR genetic* OR "dna" OR "rna" OR damag* OR insult* OR adduct* OR alkylat* OR "methylation" OR oxidizing OR oxidant* OR "oxidative stress*" OR "free radical*" OR viabilit* OR viable* OR necro* OR "necrosis" OR apopto* OR lethal* OR "surviv*" OR "adverse effect*" OR "side effect*" OR harmful* OR disease* OR illness* OR syndrome* OR symptom* OR abnormal* OR irritant* OR cancer* OR neoplas* OR tumor* OR tumour* OR malignan* OR malformat* OR anomal* OR abnormal* OR "congenital*" OR "birth defect*" OR reproduct* OR allerg* OR hypersensitiv* OR histolog* OR endocrin* OR neuroend* OR "neuro-endocrin*" OR "neurosecret*" OR physiopatho* OR pathophysi* OR patholog*)) AND (TITLE-ABS-KEY (agrochemical* OR "chemical*" OR "plant protect*" OR pesticide* OR biocid* OR herbicid* OR weedkiller* OR "weed killer*" OR defoliant* OR insecticid* OR nematicid* OR molluscicid* OR piscicid* OR avicid* OR rodenticid* OR bactericid* OR repellent* OR antimicrob* OR "antiparasit*" OR	**Results:** 3,836<br><br>**Notes:**<br>All search terms are searched in the fields: “title”, “abstract” and “keywords” (here marked with “TITLE-ABS-KEY”)<br><br>No thesaurus available.

	fungicid* OR lampricid* OR acaricid* OR miticid* OR "mite control*" OR algicid* OR algaecid* OR chemosterilant*))	

**Web of Science-Core Collection** (Clarivate)<br><br>**Coverage:** from database inception to search date<br><br>**Search date:** 2023-05-06	((((TOPIC: farmer* OR "farm" OR farms OR ranch OR rancher* OR agronomist* OR smallholder* OR grazier* OR farmhand* OR "farmers" OR agricultur* OR grower* OR reaper* OR breeder* OR cropper* OR cultivator* OR feeder* OR gardener* OR gleaner* OR harvester* OR horticulturist* OR planter* OR tiller* OR rural* OR "worker*" OR "labor*" OR "labour*") AND (TOPIC:"iraq*" OR "lebanon" OR "sudan" OR "mauritania" OR "middle east*" OR "near east" OR "east mediterranean" OR "eastern mediterran*" OR arabic OR arabs OR arab OR mena OR "arabian peninsula" OR "north africa*" OR "northern africa*" OR maghreb* OR maghrib* OR morocco* OR egypt* OR jordan* OR lebanes* OR syria* OR algeri* OR libya* OR tunis* OR uae OR "united arab*" OR emirat* OR saudi* OR ksa OR qatar* OR oman* OR yemen* OR kuwait* OR bahrain* OR palestin* OR "west bank" OR "gaza strip" OR levant* OR gulf*) AND (TOPIC:poison* OR toxic* OR genotox* OR cytotox* OR antimetabolite* OR antispermatogen* OR cardiotox* OR dermatotox* OR dermotox* OR reprotox* OR hepatotox* OR nephrotox* OR pneumotox* OR immunotox* OR neurotox* OR noxae* OR hazard* OR pharmacogenomic* OR "comet assay*" OR "micronucleus*" OR "aberrat*" OR "chromosom*" OR denaturation* OR "hybridization*" OR "ames" OR aneuploid* OR topoisomeras* OR teniposid* OR etoposid* OR greenscreen* OR "γh2ax*" OR "ph2ax*" OR "high content screening*" OR "ph3" OR "cycle arrest*" OR phospho-histon* OR phosphohiston* OR caspase* OR "tubulin microtubule*" OR "profiling assay*" OR steatosis* OR carcino* OR mutagen* OR "mutation" OR teratogen* OR disorder* OR genetic* OR "dna" OR "rna" OR damag* OR insult* OR adduct* OR alkylat* OR "methylation" OR oxidizing OR oxidant* OR "oxidative stress*" OR "free radical*" OR viabilit* OR viable* OR necro* OR "necrosis" OR apopto* OR lethal* OR "surviv*" OR "adverse effect*" OR "side effect*" OR harmful* OR disease* OR illness* OR syndrome* OR symptom* OR abnormal* OR irritant* OR cancer* OR neoplas* OR tumor* OR tumour* OR malignan* OR malformat* OR anomal* OR abnormal* OR "congenital*" OR "birth defect*" OR reproduct* OR allerg* OR hypersensitiv* OR histolog* OR endocrin* OR neuroend* OR "neuro-endocrin*" OR "neurosecret*" OR physiopatho* OR pathophysi* OR patholog*) AND (TOPIC:agrochemical* OR "chemical*" OR "plant protect*" OR pesticide* OR biocid* OR herbicid* OR weedkiller* OR "weed killer*" OR defoliant* OR insecticid* OR nematicid* OR molluscicid* OR piscicid* OR avicid* OR rodenticid* OR bactericid* OR repellent* OR antimicrob* OR "antiparasit*" OR fungicid* OR	**Results:** 2,262<br><br>**Notes:**<br>All search terms are searched in the fields: “title”, “abstract” and “keywords” (here marked with “Topic”)<br><br>No thesaurus available.

	lampricid* OR acaricid* OR miticid* OR "mite control*" OR algicid* OR algaecid* OR chemosterilant*)))) AND English OR Arabic (Languages)	
**Agricola** (EBSCOhost)<br><br>**Coverage:** from database inception to search date<br><br>**Search date:** 2023-05-06	SU ((ZU "farmer”) OR (ZU "rancher”) OR (ZU "agronomist") OR (ZU "agronomists") OR (ZU "graziers") OR (ZU "cultivator") OR (ZU "feeder") OR (ZU "breeder") OR (ZU "gleaner") OR (ZU "gleaners") OR (ZU "harvester") OR (ZU "horticulturist") OR (ZU "horticulturists") OR (ZU "planter") OR (ZU "tiller") OR (ZU "rural") OR (ZU "worker") OR (ZU "labor")) OR TI (farmer* OR "farm" OR farms OR ranch OR rancher* OR agronomist* OR smallholder* OR grazier* OR farmhand* OR "farmers" OR agricultur* OR grower* OR reaper* OR breeder* OR cropper* OR cultivator* OR feeder* OR gardener* OR gleaner* OR harvester* OR horticulturist* OR planter* OR tiller* OR rural* OR "worker*" OR "labor*" OR "labour*") OR AB (farmer* OR "farm" OR farms OR ranch OR rancher* OR agronomist* OR smallholder* OR grazier* OR farmhand* OR "farmers" OR agricultur* OR grower* OR reaper* OR breeder* OR cropper* OR cultivator* OR feeder* OR gardener* OR gleaner* OR harvester* OR horticulturist* OR planter* OR tiller* OR rural* OR "worker*" OR "labor*" OR "labour*") AND SU ((ZU "egypt") OR (ZU "lebanon") OR (ZU "iraq") OR (ZU "sudan") OR (ZU "mauritania") OR (ZU "middle east") OR (ZU "east mediterranean") OR (ZU "east mediterranean area") OR (ZU "east mediterranean region") OR (ZU "arabic") OR (ZU "arabs") OR (ZU "mena countries") OR (ZU "mena region") OR (ZU "mena region-arab countries") OR (ZU "arabian") OR (ZU "maghreb") OR (ZU "maghreb region, north africa") OR (ZU "morocco") OR (ZU "jordan") OR (ZU "lebanese") OR (ZU "syria") OR (ZU "algeria") OR (ZU "algeria & tunisia") OR (ZU "tunisia") OR (ZU "libya") OR (ZU "uae") OR (ZU "emirati population") OR (ZU "saudi") OR (ZU "saudi arabia") OR (ZU "saudi arabia (ksa)") OR (ZU "ksa") OR (ZU "qatar") OR (ZU "oman") OR (ZU "yemen") OR (ZU "kuwait") OR (ZU "bahrain") OR (ZU "palestine") OR (ZU "gulf") OR (ZU "gulf arab states") OR (ZU "west bank") OR (ZU "gaza strip") OR (ZU "levant")) OR TI ("iraq*" OR "lebanon" OR "Sudan" OR "Mauritania" OR "middle east*" OR "near east" OR "east mediterranean" OR "eastern mediterran*" OR arabic OR arabs OR arab OR mena OR "arabian peninsula" OR "north africa*" OR "northern africa*" OR maghreb* OR maghrib* OR morocco* OR egypt* OR jordan* OR lebanes* OR syria* OR algeri* OR libya* OR tunis* OR uae OR "united arab*" OR emirat* OR saudi* OR ksa OR qatar* OR oman* OR yemen* OR kuwait* OR bahrain* OR palestin* OR "west bank" OR "gaza strip" OR levant* OR gulf*) OR AB ("iraq*" OR "lebanon" OR "Sudan" OR "Mauritania" OR "middle east*" OR "near east" OR "east mediterranean" OR "eastern mediterran*" OR arabic OR arabs OR arab OR mena OR "arabian peninsula" OR "north africa*" OR "northern africa*" OR maghreb* OR maghrib* OR morocco* OR egypt* OR jordan* OR lebanes* OR syria* OR algeri* OR libya* OR tunis* OR uae OR "united arab*" OR emirat* OR saudi* OR ksa OR qatar* OR oman* OR yemen* OR kuwait* OR bahrain* OR	**Results:** 1,558<br><br>**Notes:**<br>All search terms are searched in the “title”, “abstract” and in the “subject” fields (here marked as: “TI”, “AB” & “SU”)<br><br>thesaurus was used by subject headings.<br><br>Filters for English and Arabic languages are applied.

	palestin* OR "west bank" OR "gaza strip" OR levant* OR gulf*) AND SU ((ZU "poison") OR (ZU "toxic") OR (ZU "genotoxic") OR (ZU "genotoxic agents") OR (ZU "cytotoxic") OR (ZU "cytotoxic activity") OR (ZU "cytotoxic agents") OR (ZU "antimetabolite") OR (ZU "reprotoxic") OR (ZU "reprotoxicity") OR (ZU "cardiotoxic") OR (ZU "cardiotoxicity") OR (ZU "dermatoxin") OR (ZU "hazard") OR (ZU "hazard & risk") OR (ZU "comet assay") OR (ZU "comet assays") OR (ZU "pneumotoxicity") OR (ZU "neurotox") OR (ZU "neurotoxic") OR (ZU "neurotoxic agents") OR (ZU "micronucleus") OR (ZU "micronucleus mn test") OR (ZU "micronucleus and comet assays") OR (ZU "pharmacogenomic") OR (ZU "pharmacogenomics") OR (ZU "chromosomal") OR (ZU "chromosomal aberration") OR (ZU "chromosomal aberration ca test") OR (ZU "chromosomal aberration assay") OR (ZU "chromosomal aberration test") OR (ZU "chromosomal aberrations") OR (ZU "chromosomal abnormalities") OR (ZU "chromosomal anomalies") OR (ZU "denaturation") OR (ZU "hybridization, genetic") OR (ZU "hybridization") OR (ZU "ames-test") OR (ZU "aneuploid") OR (ZU "aneuploidy") OR (ZU "topoisomerase") OR (ZU "teniposide") OR (ZU "etoposide") OR (ZU "greenscreen") OR (ZU "γh2ax") OR (ZU "mutagen") OR (ZU "mutagenesis") OR (ZU "carcinogen") OR (ZU "disorder-based functions") OR (ZU "genetic") OR (ZU "caspase") OR (ZU "damage") OR (ZU "oxidative stress") OR (ZU "free radical") OR (ZU "adverse effect") OR (ZU "adverse effects") OR (ZU "teratogen") OR (ZU "teratogenesis") OR (ZU "apoptosis") OR (ZU "lethal") OR (ZU "necrosis") OR (ZU "dna") OR (ZU "rna") OR (ZU "survival") OR (ZU "abnormal") OR (ZU "cancer") OR (ZU "tumor") OR (ZU "side effect") OR (ZU "side effects") OR (ZU "irritant") OR (ZU "irritant agents") OR (ZU "hypersensitive cell death") OR (ZU "hypersensitive reaction") OR (ZU "illness") OR (ZU "syndrome") OR (ZU "disease") OR (ZU "birth defect") OR (ZU "birth defects") OR (ZU "congenital") OR (ZU "congenital abnormalities") OR (ZU "symptom") OR (ZU "physiopathology") OR (ZU "physiopathy") OR (ZU "patho-epidemiology") OR (ZU "pathologic") OR (ZU "hypersensitive reaction") OR (ZU "hypersensitive response")) OR TI (poison* OR toxic* OR genotox* OR cytotox* OR antimetabolite* OR antispermatogen* OR cardiotox* OR dermatotox* OR dermotox* OR reprotox* OR hepatotox* OR nephrotox* OR pneumotox* OR immunotox* OR neurotox* OR noxae* OR hazard* OR pharmacogenomic* OR "comet assay*" OR "micronucleus*" OR "aberrat*" OR "chromosom*" OR denaturation* OR "hybridization*" OR "ames" OR aneuploid* OR topoisomeras* OR teniposid* OR etoposid* OR greenscreen* OR "γh2ax*" OR "ph2ax*" OR "high content screening*" OR "ph3" OR "cycle arrest*" OR phospho-histon* OR phosphohiston* OR caspase* OR "tubulin microtubule*" OR "profiling assay*" OR steatosis* OR carcino* OR mutagen* OR "mutation" OR teratogen* OR disorder* OR genetic* OR "dna" OR "rna" OR damag* OR insult* OR adduct* OR alkylat* OR "methylation" OR oxidizing OR oxidant* OR "oxidative stress*" OR "free radical*" OR	

	viabilit* OR viable* OR necro* OR "necrosis" OR apopto* OR lethal* OR "surviv*" OR "adverse effect*" OR "side effect*" OR harmful* OR disease* OR illness* OR syndrome* OR symptom* OR abnormal* OR irritant* OR cancer* OR neoplas* OR tumor* OR tumour* OR malignan* OR malformat* OR anomal* OR abnormal* OR "congenital*" OR "birth defect*" OR reproduct* OR allerg* OR hypersensitiv* OR histolog* OR endocrin* OR neuroend* OR "neuro-endocrin*" OR "neurosecret*" OR physiopatho* OR pathophysi* OR patholog*) OR AB (poison* OR toxic* OR genotox* OR cytotox* OR antimetabolite* OR antispermatogen* OR cardiotox* OR dermatotox* OR dermotox* OR reprotox* OR hepatotox* OR nephrotox* OR pneumotox* OR immunotox* OR neurotox* OR noxae* OR hazard* OR pharmacogenomic* OR "comet assay*" OR "micronucleus*" OR "aberrat*" OR "chromosom*" OR denaturation* OR "hybridization*" OR "ames" OR aneuploid* OR topoisomeras* OR teniposid* OR etoposid* OR greenscreen* OR "γh2ax*" OR "ph2ax*" OR "high content screening*" OR "ph3" OR "cycle arrest*" OR phospho-histon* OR phosphohiston* OR caspase* OR "tubulin microtubule*" OR "profiling assay*" OR steatosis* OR carcino* OR mutagen* OR "mutation" OR teratogen* OR disorder* OR genetic* OR "dna" OR "rna" OR damag* OR insult* OR adduct* OR alkylat* OR "methylation" OR oxidizing OR oxidant* OR "oxidative stress*" OR "free radical*" OR viabilit* OR viable* OR necro* OR "necrosis" OR apopto* OR lethal* OR "surviv*" OR "adverse effect*" OR "side effect*" OR harmful* OR disease* OR illness* OR syndrome* OR symptom* OR abnormal* OR irritant* OR cancer* OR neoplas* OR tumor* OR tumour* OR malignan* OR malformat* OR anomal* OR abnormal* OR "congenital*" OR "birth defect*" OR reproduct* OR allerg* OR hypersensitiv* OR histolog* OR endocrin* OR neuroend* OR "neuro-endocrin*" OR "neurosecret*" OR physiopatho* OR pathophysi* OR patholog*) AND SU ((ZU "agrochemical") OR (ZU "chemical") OR (ZU "plant protection") OR (ZU "pesticide") OR (ZU "pesticide action") OR (ZU "pesticide adjuvants") OR (ZU "biocidal") OR (ZU "herbicid") OR (ZU "herbicidal action") OR (ZU "weedkillers") OR (ZU "weed killer") OR (ZU "weed killers") OR (ZU "defoliant") OR (ZU "defoliant alternatives") OR (ZU "defoliants") OR (ZU "defoliating insects") OR (ZU "insecticidal") OR (ZU "insecticidal action") OR (ZU "insecticidal activities") OR (ZU "insecticidal activity") OR (ZU "nematicidal") OR (ZU "nematicidal activity") OR (ZU "nematicidal agent") OR (ZU "molluscicidal") OR (ZU "molluscicidal activity") OR (ZU "molluscicidal agents") OR (ZU "molluscicidal drugs") OR (ZU "piscicidal activity") OR (ZU "piscicidal plant") OR (ZU "piscicidal plants") OR (ZU "avicide") OR (ZU "avicides") OR (ZU "rodenticidal plants") OR (ZU "rodenticide") OR (ZU "rodenticide application") OR (ZU "rodenticides") OR (ZU "bactericida") OR (ZU "bactericidal") OR (ZU "bactericidal activity") OR (ZU "bactericidal agent") OR (ZU "repellent") OR (ZU "repellent activity") OR (ZU "repellent and antinematodal activities") OR (ZU "repellent and insecticide activity")	

	OR (ZU "antiparasitaires") OR (ZU "antiparasite") OR (ZU "antiparasitic") OR (ZU "antiparasitic agents") OR (ZU "fungicidal") OR (ZU "fungicidal activity") OR (ZU "lampricide") OR (ZU "lampricide toxicity") OR (ZU "lampricides") OR (ZU "acaricidal") OR (ZU "acaricidal agents") OR (ZU "acaricide") OR (ZU "miticide") OR (ZU "miticide residues") OR (ZU "miticides") OR (ZU "miticides") OR (ZU "mite control") OR (ZU "algicidal") OR (ZU "algaecide") OR (ZU "algaecides") OR (ZU "chemosterilants")) OR TI (agrochemical* OR "chemical*" OR "plant protect*" OR pesticide* OR biocid* OR herbicid* OR weedkiller* OR "weed killer*" OR defoliant* OR insecticid* OR nematicid* OR molluscicid* OR piscicid* OR avicid* OR rodenticid* OR bactericid* OR repellent* OR antimicrob* OR "antiparasit*" OR fungicid* OR lampricid* OR acaricid* OR miticid* OR "mite control*" OR algicid* OR algaecid* OR chemosterilant*) OR AB (agrochemical* OR "chemical*" OR "plant protect*" OR pesticide* OR biocid* OR herbicid* OR weedkiller* OR "weed killer*" OR defoliant* OR insecticid* OR nematicid* OR molluscicid* OR piscicid* OR avicid* OR rodenticid* OR bactericid* OR repellent* OR antimicrob* OR "antiparasit*" OR fungicid* OR lampricid* OR acaricid* OR miticid* OR "mite control*" OR algicid* OR algaecid* OR chemosterilant*)	

**The Index Medicus for the Eastern Mediterranean Region-IMEMR** (WHO)<br><br>**Coverage:** from database inception to search date<br><br>**Search date:** 2023-05-06<br><br>**Access through:** pesquisa.bvsalud.org/	tw:((ab:(poison* OR toxic* OR genotox* OR cytotox* OR antimetabolite* OR antispermatogen* OR cardiotox* OR dermatotox* OR dermotox* OR reprotox* OR hepatotox* OR nephrotox* OR pneumotox* OR immunotox* OR neurotox* OR noxae* OR hazard* OR pharmacogenomic* OR "comet assay*" OR "micronucleus*" OR "aberrat*" OR "chromosom*" OR denaturation* OR "hybridization*" OR "ames" OR aneuploid* OR topoisomeras* OR teniposid* OR etoposid* OR greenscreen* OR "γh2ax*" OR "ph2ax*" OR "high content screening*" OR "ph3" OR "cycle arrest*" OR phospho-histon* OR phosphohiston* OR caspase* OR "tubulin microtubule*" OR "profiling assay*" OR steatosis* OR carcino* OR mutagen* OR "mutation" OR teratogen* OR disorder* OR genetic* OR "dna" OR "rna" OR damag* OR insult* OR adduct* OR alkylat* OR "methylation" OR oxidizing OR oxidant* OR "oxidative stress*" OR "free radical*" OR viabilit* OR viable* OR necro* OR "necrosis" OR apopto* OR lethal* OR "surviv*" OR "adverse effect*" OR "side effect*" OR harmful* OR disease* OR illness* OR syndrome* OR symptom* OR abnormal* OR irritant* OR cancer* OR neoplas* OR tumor* OR tumour* OR malignan* OR malformat* OR anomal* OR abnormal* OR "congenital*" OR "birth defect*" OR reproduct* OR allerg* OR hypersensitiv* OR histolog* OR endocrin* OR neuroend* OR "neuro-endocrin*" OR "neurosecret*" OR physiopatho* OR pathophysi* OR patholog*)) AND (ab:("iraq*" OR "lebanon" OR "sudan" OR "mauritania" OR "middle east*" OR "near east" OR "east mediterranean" OR "eastern mediterran*" OR arabic OR arabs OR arab OR mena OR "arabian peninsula" OR "north africa*" OR "northern africa*" OR maghreb* OR maghrib* OR morocco* OR egypt* OR jordan* OR lebanes* OR syria* OR algeri* OR libya* OR tunis* OR uae OR "united arab*" OR emirat* OR saudi* OR ksa OR qatar* OR oman* OR yemen* OR kuwait* OR bahrain* OR palestin* OR "west bank" OR "gaza strip" OR levant* OR gulf*)) AND (ab:(farmer* OR "farm" OR farms OR ranch OR rancher* OR agronomist* OR smallholder* OR grazier* OR farmhand* OR "farmers" OR agricultur* OR grower* OR reaper* OR breeder* OR cropper* OR cultivator* OR feeder* OR gardener* OR gleaner* OR harvester* OR horticulturist* OR planter* OR tiller* OR rural* OR "worker*" OR "labor*" OR "labour*")) AND (ab:(agrochemical* OR "chemical*" OR "plant protect*" OR pesticide* OR biocid* OR herbicid* OR weedkiller* OR "weed killer*" OR defoliant* OR insecticid* OR nematicid* OR molluscicid* OR piscicid* OR avicid* OR rodenticid* OR bactericid* OR repellent* OR antimicrob* OR	**Results:** 435<br><br>**Notes:**<br>All search terms are searched in the fields: “abstract” (here marked with “ab”)<br><br>No thesaurus available.

	"antiparasit*" OR fungicid* OR lampricid* OR acaricid* OR miticid* OR "mite control*" OR algicid* OR algaecid* OR chemosterilant*))) AND (collection_gim:("IMEMR") AND la:("en" OR "ar"))	

**EMBASE** (Elsevier)<br><br>**Source included: Embase only**<br><br>**Coverage:** from database inception to search date<br><br>**Search date:** 2023-05-06	((('gardener'/de OR 'agricultural worker'/de OR 'worker'/de OR farmer*:ti,ab,kw OR 'farm':ti,ab,kw OR farms:ti,ab,kw OR ranch:ti,ab,kw OR rancher*:ti,ab,kw OR agronomist*:ti,ab,kw OR smallholder*:ti,ab,kw OR grazier*:ti,ab,kw OR farmhand*:ti,ab,kw OR agricultur*:ti,ab,kw OR grower*:ti,ab,kw OR reaper*:ti,ab,kw OR breeder*:ti,ab,kw OR cropper*:ti,ab,kw OR cultivator*:ti,ab,kw OR feeder*:ti,ab,kw OR gardener*:ti,ab,kw OR gleaner*:ti,ab,kw OR harvester*:ti,ab,kw OR horticulturist*:ti,ab,kw OR planter*:ti,ab,kw OR tiller*:ti,ab,kw OR rural*:ti,ab,kw OR 'worker*':ti,ab,kw OR 'labor*':ti,ab,kw OR 'labour*':ti,ab,kw) AND ('syrian arab republic'/de OR 'egypt'/de OR 'lebanon'/de OR 'united arab emirates'/de OR 'jordan'/de OR 'saudi arabia'/de OR 'morocco'/de OR 'sudan'/de OR 'algeria'/de OR 'yemen'/de OR 'kuwait'/de OR 'bahrain'/de OR 'iraq'/de OR 'oman'/de OR 'north africa'/de OR 'middle east'/de OR 'arabic'/de OR 'arab'/de OR 'qatar'/de OR 'libyan arab jamahiriya'/de OR 'tunisia'/de OR 'palestine'/de OR 'gaza strip'/de OR 'mauritania'/de OR 'iraq*':ti,ab,kw OR 'lebanon':ti,ab,kw OR 'sudan':ti,ab,kw OR 'mauritania':ti,ab,kw OR 'middle east*':ti,ab,kw OR 'near east':ti,ab,kw OR 'east mediterranean':ti,ab,kw OR 'eastern mediterran*':ti,ab,kw OR arabic:ti,ab,kw OR arabs:ti,ab,kw OR arab:ti,ab,kw OR mena:ti,ab,kw OR 'arabian peninsula':ti,ab,kw OR 'north africa*':ti,ab,kw OR 'northern africa*':ti,ab,kw OR maghreb*:ti,ab,kw OR maghrib*:ti,ab,kw OR morocco*:ti,ab,kw OR egypt*:ti,ab,kw OR jordan*:ti,ab,kw OR lebanes*:ti,ab,kw OR syria*:ti,ab,kw OR algeri*:ti,ab,kw OR libya*:ti,ab,kw OR tunis*:ti,ab,kw OR uae:ti,ab,kw OR 'united arab*':ti,ab,kw OR emirat*:ti,ab,kw OR saudi*:ti,ab,kw OR ksa:ti,ab,kw OR qatar*:ti,ab,kw OR oman*:ti,ab,kw OR yemen*:ti,ab,kw OR kuwait*:ti,ab,kw OR bahrain*:ti,ab,kw OR palestin*:ti,ab,kw OR 'west bank':ti,ab,kw OR 'gaza strip':ti,ab,kw OR levant*:ti,ab,kw OR gulf*:ti,ab,kw) AND ('poison'/de OR 'toxic substance'/de OR 'genotoxicity assay'/de OR 'genotoxicity'/de OR 'cytotoxicity'/de OR 'cytotoxicity assay'/de OR 'antimetabolite'/de OR 'antispermatogenic agent'/de OR 'cardiotoxin'/de OR 'hepatotoxin'/exp OR 'hepatotoxic agent'/de OR 'nephrotoxicity'/de OR 'immunotoxin'/de OR 'neurotoxin'/de OR 'hazard'/de OR 'pharmacogenomics'/de OR 'comet assay'/de OR 'micronucleus'/de OR 'micronucleus test'/de OR 'chromosome aberration'/de OR 'chromosome'/de OR 'denaturation'/exp OR 'hybridization'/de OR 'ames test'/de OR 'aneuploidy'/de OR 'dna topoisomerase'/de OR 'teniposide'/de OR 'etoposide'/de OR 'cell cycle arrest'/de OR 'caspase'/de OR 'carcinoma'/de OR 'metastasis'/de OR 'mutagenic	**Results:** 1,386<br><br>**Notes:**<br>All search terms are searched in the fields: “title”, “abstract”, “Author keywords” (here marked with “:ab,ti,kw”)<br><br>The thesaurus available through Emtree.

	agent'/de OR 'teratogenic agent'/de OR 'diseases'/de OR 'genetic'/de OR 'dna'/de OR 'rna'/de OR 'dna damage'/de OR 'insult'/de OR 'alkylating agent'/de OR 'methylation'/de OR 'oxidizing agent'/de OR 'oxidative stress'/de OR 'free radical'/de OR 'cell viability'/de OR 'cell viability rate'/de OR 'necrosis'/de OR 'apoptosis'/de OR 'lethal'/de OR 'lethality'/de OR 'survival'/de OR 'adverse event'/de OR 'side effect'/de OR 'syndrome'/de OR 'symptom'/de OR 'irritant agent'/de OR 'malignant neoplasm'/de OR 'congenital malformation'/de OR 'reproduction'/de OR 'immediate type hypersensitivity'/de OR 'pathophysiology'/de OR poison*:ti,ab,kw OR toxic*:ti,ab,kw OR genotox*:ti,ab,kw OR cytotox*:ti,ab,kw OR antimetabolite*:ti,ab,kw OR antispermatogen*:ti,ab,kw OR cardiotox*:ti,ab,kw OR dermatotox*:ti,ab,kw OR dermotox*:ti,ab,kw OR reprotox*:ti,ab,kw OR hepatotox*:ti,ab,kw OR nephrotox*:ti,ab,kw OR pneumotox*:ti,ab,kw OR immunotox*:ti,ab,kw OR neurotox*:ti,ab,kw OR noxae*:ti,ab,kw OR hazard*:ti,ab,kw OR pharmacogenomic*:ti,ab,kw OR 'comet assay*':ti,ab,kw OR 'micronucleus*':ti,ab,kw OR 'aberrat*':ti,ab,kw OR 'chromosom*':ti,ab,kw OR denaturation*:ti,ab,kw OR 'hybridization*':ti,ab,kw OR 'ames':ti,ab,kw OR aneuploid*:ti,ab,kw OR topoisomeras*:ti,ab,kw OR teniposid*:ti,ab,kw OR etoposid*:ti,ab,kw OR greenscreen*:ti,ab,kw OR 'γh2ax*':ti,ab,kw OR 'ph2ax*':ti,ab,kw OR 'high content screening*':ti,ab,kw OR 'ph3':ti,ab,kw OR 'cycle arrest*':ti,ab,kw OR 'phospho histon*':ti,ab,kw OR phosphohiston*:ti,ab,kw OR caspase*:ti,ab,kw OR 'tubulin microtubule*':ti,ab,kw OR 'profiling assay*':ti,ab,kw OR steatosis*:ti,ab,kw OR carcino*:ti,ab,kw OR mutagen*:ti,ab,kw OR 'mutation':ti,ab,kw OR teratogen*:ti,ab,kw OR disorder*:ti,ab,kw OR genetic*:ti,ab,kw OR 'dna':ti,ab,kw OR 'rna':ti,ab,kw OR damag*:ti,ab,kw OR insult*:ti,ab,kw OR adduct*:ti,ab,kw OR alkylat*:ti,ab,kw OR 'methylation':ti,ab,kw OR oxidizing:ti,ab,kw OR oxidant*:ti,ab,kw OR 'oxidative stress*':ti,ab,kw OR 'free radical*':ti,ab,kw OR viabilit*:ti,ab,kw OR viable*:ti,ab,kw OR necro*:ti,ab,kw OR 'necrosis':ti,ab,kw OR apopto*:ti,ab,kw OR lethal*:ti,ab,kw OR 'surviv*':ti,ab,kw OR 'adverse effect*':ti,ab,kw OR 'side effect*':ti,ab,kw OR harmful*:ti,ab,kw OR disease*:ti,ab,kw OR illness*:ti,ab,kw OR syndrome*:ti,ab,kw OR symptom*:ti,ab,kw OR irritant*:ti,ab,kw OR cancer*:ti,ab,kw OR neoplas*:ti,ab,kw OR tumor*:ti,ab,kw OR tumour*:ti,ab,kw OR malignan*:ti,ab,kw OR malformat*:ti,ab,kw OR anomal*:ti,ab,kw OR abnormal*:ti,ab,kw OR 'congenital*':ti,ab,kw OR 'birth defect*':ti,ab,kw OR	

	reproduct*:ti,ab,kw OR allerg*:ti,ab,kw OR hypersensitiv*:ti,ab,kw OR histolog*:ti,ab,kw OR endocrin*:ti,ab,kw OR neuroend*:ti,ab,kw OR 'neuro-endocrin*':ti,ab,kw OR 'neurosecret*':ti,ab,kw OR physiopatho*:ti,ab,kw OR pathophysi*:ti,ab,kw OR patholog*:ti,ab,kw) AND ('agricultural chemical'/de OR 'chemical compound'/de OR 'plant protection product'/de OR 'pesticide'/exp OR 'chemosterilant'/de OR 'acaricide'/de OR 'algaecide'/de OR 'pest control'/exp OR agrochemical*:ti,ab,kw OR 'chemical*':ti,ab,kw OR 'plant protect*':ti,ab,kw OR pesticide*:ti,ab,kw OR biocid*:ti,ab,kw OR herbicid*:ti,ab,kw OR weedkiller*:ti,ab,kw OR 'weed killer*':ti,ab,kw OR defoliant*:ti,ab,kw OR insecticid*:ti,ab,kw OR nematicid*:ti,ab,kw OR molluscicid*:ti,ab,kw OR piscicid*:ti,ab,kw OR avicid*:ti,ab,kw OR rodenticid*:ti,ab,kw OR bactericid*:ti,ab,kw OR repellent*:ti,ab,kw OR antimicrob*:ti,ab,kw OR 'antiparasit*':ti,ab,kw OR fungicid*:ti,ab,kw OR lampricid*:ti,ab,kw OR acaricid*:ti,ab,kw OR miticid*:ti,ab,kw OR 'mite control*':ti,ab,kw OR algicid*:ti,ab,kw OR algaecid*:ti,ab,kw OR chemosterilant*:ti,ab,kw)))AND ([arabic]/lim OR [english]/lim)	
